# Study on Exosomes Promoting the Osteogenic Differentiation of ADSCs in Graphene Porous Titanium Alloy Scaffolds

**DOI:** 10.3389/fbioe.2022.905511

**Published:** 2022-06-06

**Authors:** Xu Sun, Shude Yang, Shuang Tong, Shu Guo

**Affiliations:** Department of Plastic Surgery, The First Hospital of China Medical University, Shenyang, China

**Keywords:** bone tissue engineering, exosomes, titanium alloy, graphene, adiposederived stem cells

## Abstract

Titanium and titanium alloys (Ti_6_Al_4_V and Ti) have been widely used in bone tissue engineering to repair maxillofacial bone defects caused by traumas and tumors. However, such materials are also bio-inert, which does not match the elastic modulus of bone. Therefore, different surface modifications have been proposed for clinical application. Based on the use of traditional titanium alloy in the field of bone repair defects, we prepared a compound Gr-Ti scaffold with ADSC-derived Exos. The results showed that Gr-Ti scaffolds have low toxicity and good biocompatibility, which can promote the adhesion and osteogenic differentiation of ADSCs. Exos played a role in promoting osteogenic differentiation of ADSCs: the mRNA levels of *RUNX2*, *ALP*, and *Osterix* in the Gr-Ti/Exos group were significantly higher than those in the Gr-Ti group, which process related to the Wnt signaling pathway. Gr-Ti scaffolds with ADSCs and ADSC-derived Exos successfully repaired rabbit mandibular defects. The bone mineral density and the bending strength of the Gr-Ti/Exos group was significantly higher than that of the Gr-Ti group. This study provides a theoretical basis for the research and development of new clinical bone repair materials.

## 1 Introduction

Tissue engineering technology has opened up new ways to address jaw defects (Altaie et al., 2016). To repair bone defects, porous tissue engineering scaffolds were designed to guide the proliferation of the cells of a patient on the corresponding surface, and thus realize the repair of bone trauma and reconstruction of bone defects (Azadian et al., 2020; Mitra et al., 2021).

Titanium alloys are the most commonly used metals, with successful applications in plastic surgery, orthopedic, and dental implants (Gruenwald et al., 2018; Fan et al., 2022). However, titanium alloy materials are mostly solid structures, and the high elastic modulus of titanium alloy materials produces stress shielding effects on the bone tissue (Tamayo et al., 2021). Therefore, different surface modification techniques have been proposed to meet the clinical requirements. The common surface modification methods of implants mainly include two aspects: one is the loose treatment of implant structure; Second, implant surface coating. In terms of structure, porous scaffolds can be constructed by 3D printing, and graphene materials can be considered as the surface coating. Since its discovery, graphene has also been extended to biomedical applications (Pereira et al., 2021). Graphene modification can improve the osteogenic differentiation of stem cells (Lu et al., 2020; Wu et al., 2020). In our previous study, we produced porous titanium alloy scaffolds with low elastic modulus and graphene coating by selective laser melting (SLM) and micro-arc oxidation (MAO) technology.

Adipose stem cells (ADSCs) are mesenchymal stem cells (MSCs) isolated from adipose tissue (Bunnell, 2021). In recent years, numerous studies have been performed on ADSCs in the field of bone regeneration which confirmed that ADSCs have the potential to differentiate into adipocytes, osteoblasts, and chondrocytes. Recently, increasing studies have shown that the therapeutic effect of ADSCs is not only related to its differentiation ability, but also its paracrine action (Ajit and Ambika Gopalankutty, 2021). Exosomes (Exos) are spherical extracellular vesicles (EVs) with a diameter of 30–150 nm and a lipid bilayer structure (Lai et al., 2015). Exos can deliver specific proteins, miRNAs, and cytokines to regulate stem cell differentiation and activate related signaling pathways to promote bone repair (Tang et al., 2021). Therefore, it is necessary to study the internal mechanism of Exos in bone regeneration as well as explore methods of preparing Exos and tissue engineering scaffold composite materials for bone tissue engineering.

In this study, Exos acted as an inducer to promote the adhesion, proliferation, and osteogenic differentiation of ADSCs on Gr-Ti scaffolds. The expression of osteogenic genes and proteins was detected by PCR analysis and western blot. Specific focus was placed on exploring the key protein levels of the Wnt signaling pathway to provide theoretical and experimental data for clinical experiments and applications. Furthermore, a critical bone defect model was established in the mandible of New Zealand white rabbits, and the effects of ADSCs, Exos, and composite implants on bone repair were evaluated through gross observation, imaging analysis, and histological examination. This study aims to provide a theoretical basis for the combined application of bone tissue engineering and stem cell therapy.

## 2 Materials and Methods

### 2.1 Preparation and Characterization of Materials

Porous titanium alloys with external shapes of 8 mm × 4 mm × 3 mm and 4 mm × 4 mm × 3 mm were designed by using CAD v23.0 (Materialise, Leuven, Belgium). The internal pore parameters were designed by taking a cube as the basic structural unit and the aperture size was designed as 550 μm. The porosity was ∼70%. The two sizes of porous titanium alloy scaffolds were prepared by SLM technology. The scaffold was connected with wires after pretreatment and placed into the prepared electrolyte as required. The graphite plate and the support were set as anode and cathode, and the corresponding electrolyte group was divided into 4 g/L graphene (Nanjing XFNANO Materials Tech Co. Ltd., China) and 5 g/L EDTA (Wujiang Aobang Chemical Co. Ltd., China). Oxidation treatment was performed under constant current (2 A/dm^2^ mode for 30 min) and the preparation of graphene coating was completed by MAO. The surface morphology of Ti and Gr-Ti obtained by micro-arc oxidation technology was observed by scanning electron microscopy (JSM-TM3000, Japan).

### 2.2 Extraction and Identification of Exos Derived From Adipose Stem Cells

The adipose tissue used in this study was obtained from patients undergoing liposuction. Patients with systemic diseases such as hepatitis, metabolic diseases, HIV, syphilis, or cancer were excluded. All patients signed informed consents and received consent from the ETHICS committee.

Human adipose tissue was obtained under aseptic conditions and 0.2% type I collagenase (Sigma Chemical Co., St. Louis, MO, United States) was used for full digestion in a 37°C water bath for 45 min. Supernatant and adipose tissue were discarded after centrifugation at 1,200 rpm for 5 min at room temperature. The cells were resuspended by blowing with 15% FBS (Gibco, United States) and inoculated in a 75 cm^2^ culture flask at 37°C with 5% CO_2_ saturated humidity.

The ADSC culture medium from the third to fifth generations was collected and Exos were extracted by differential ultracentrifugation. Centrifugation at 4°C for 10 min at low speed, and the supernatant was collected. The supernatant was then centrifuged at 2,000 × g for 20 min. The supernatant was transferred to another centrifuge tube and centrifuged at room temperature for 30 min at high speed. After centrifugation for 70 min at 130,000 × g, the precipitates were collected, which contained exosomes and impurities. Then PBS was used for re-suspension and centrifugation again.

Exos were observed by transmission electron microscopy (Hitachi, Japan), and the exosome-specific surface proteins TSG101 and CD9 were detected by western blot.

### 2.3 Co-Culturing Cells With Scaffold Materials

Preliminary experimental results showed that the ability of Gr-Ti scaffolds to promote cell adhesion and proliferation was superior to Ti scaffolds. Therefore, only the relationship between Exos and Gr-Ti scaffolds was studied in this study. The experimental groups consisted of a blank control group, Gr-Ti group, and Gr-Ti/Exos group.

The support material was sterilized at high temperature and high pressure for later use. Before use, the support material was washed three times with PBS and placed in a 96-well plate. The density of the ADSCs was adjusted to 1 × 10^5^/ml, and then inoculated into culture plates and fully cultured at 37°C and 5% CO_2_. The Gr-Ti/Exos group was supplemented with 50 μl/well Exos.

#### 2.3.1 Early Growth of Cells in the Scaffold Detected by SEM

The samples were taken at 4 and 24 h. The morphology was observed under a microscope and photographed.

#### 2.3.2 CCK-8 Toxicity Test

The cell suspension was diluted, and the corresponding density was controlled to reach 1 × 10^6^/ml. Then, it was inoculated into 96-well plates, and 0.1 ml diluted suspension was added to each well. After incubation at 25°C for 24 h, a culture medium containing CCK-8 (Beyotime, China) was added, and absorbance was measured at 450 nm (MD SpectraMax Plus 384).

#### 2.3.3 ALP Activity Determination

The detected cells were lysed by Cell lysis buffer for Western and IP without inhibitors (Beyotime Biotechnology, China), and the supernatant was collected for semi-quantitative analysis of ALP using an Alkaline Phosphatase Assay Kit (Beyotime iotechnology, China) according to the manufacturers’ instructions. Optical density (OD) values were measured using a microplate reader (SpectraMax Plus384, Molecular Devices, United States) at 405 nm.

#### 2.3.4 Quantitative Real-Time PCR

To detect the expression of the *RUNX2*, *ALP*, and *OSX* genes related to Exos promoting the osteogenic differentiation of ADSCs, Quantitative Real-Time PCR (qPCR) was used to detect the levels of related mRNA at 7 and 14 days of incubation. TRIzol™ Reagent (Invitrogen, United States) and PrimeScript™ RT Master Mix (TAKARA, Japan) were used to extract total RNA and synthesize cDNA from cells, respectively. The cycle was as follows: pre-denaturation at 95°C for 30 s; denaturation at 95°C for 5 s; and extension at 60°C for 30 s. The cycle was done for 40 rounds. All primers were synthesized by GenePharma (China). The primer sequences are given in Table 1.

**TABLE 1:** List of gene primers.

Gene	Forward sequence	Reverse sequence
RUNX2	CCG​CCT​CAG​TGA​TTT​AGG​GC	GGG​TCT​GTA​ATC​TGA​CTC​TGT​CC
ALP	AAT​CGG​GCG​TCC​AGA​CAA​C	GAG​CCT​GGG​GAT​GTT​CCT​TC
Osterix	GAG​GCA​ACT​GGC​TAG​GTG​G	TGA​GGG​CTC​CTA​GCG​GTT​TA
β-cactin	TCA​CCA​TGG​ATG​ATG​ATA​TCG​C	CTG​GAT​TAA​GGG​GAG​CAA​AGT​C

#### 2.3.5 Western Blot Analysis

Cells were lysed by RIPA Lysis Buffer (Beyotime Biotechnology, China). The proteins are electrophoretic separated with 11% SDS-PAGE gel, transferred to PVDF membrane (Millipore, United States) and stained with Ponceau S staining solution (Beyotime Biotechnology, China) for 5–10 min. After blocking with 5% evaporated skimmed milk, the membranes were incubated with each primary antibody, including anti-TSG101, anti-Calnexin, anti-RUNX2, anti-Osterix, anti-Wnt1, anti-β-catenin, and anti-Axin2 (Abcam, 1:1,000 dilution) for 16 h and the respective secondary antibody (Cell Signaling Technology, 1:5,000 dilution). After the membranes were washed with TBST three times, the target bands were detected by ECL kit (Solarbio, China).

### 2.4 *In Vivo* Study of Bone Defects by Gr-Ti Scaffold and Exosomes

#### 2.4.1 Preparation of Rabbit Mandibular Defect Model

ADSCs were isolated and cultured from inguinal adipose tissue. The Exos derived from ADSCs were extracted by differential centrifugation. The specific method is the same as was used in the previous part of the study. The experimental animals were randomly divided into three groups (6 animals/group): control, Gr-Ti, and Gr-Ti/Exos.

After the experimental animals were weighed, Zoletil (Tiletamine and Zolazepam) was injected intravenously for anesthesia. The skin was prepared on both sides of the submaxillary area and neck area. A transverse incision with a length of ∼3 cm was made along the lower edge of the mandible. The skin was cut to the bone surface, and the mandible was separated and exposed. For the Gr-Ti group, a cell suspension with a concentration of 1 × 10^5^ ADSCs/well was added to a 24-well plate. For the Gr-Ti/Exos group, a cell suspension with a concentration of 1 × 10^5^ ADSCs and 50 μl Exos was added to each well. After 24 h co-culture, scaffold materials, ADSCs, and Exos were implanted according to the pre-operative grouping, and the wound was tightly sutured.

#### 2.4.2 Observation of Experimental Results *In Vivo*

##### 2.4.2.1 General Observation

After sampling, the combination of the scaffold and surrounding tissues was observed, and the adhesion of fibrous granulation and scaffold prolapse were judged.

##### 2.4.2.2 Micro-CT Examination

Micro-CT examination was performed at 4 and 12 weeks post-operatively to evaluate the position of the scaffold and the repair of the surrounding bone defect area, respectively.

##### 2.4.2.3 Bone Density Measurement

At 4 and 12 weeks post-operatively, the bone mineral density was measured by a dual-energy X-ray bone density instrument (Aishen Technology Development, Shanghai, China).

##### 2.4.2.4 Biomechanical Determination

After the bone mineral density was measured post-operatively at 4 and 12 weeks, the biomechanical properties of the material were tested using an universal mechanics testing machine (Zwick, Germany).

##### 2.4.2.5 Histological Observation

Samples were taken from the bone defect area at 4 and 12 weeks post-operation, and Van Gieson staining was used for observation and photography.

### 2.5 Statistical Analysis

All experiments were repeated at least three times before statistical analyses. All statistical analyses were performed with SPSS v19.0. Differences between groups were analyzed using a one-way analysis of variance (ANOVA) followed by Tukey’s test.

## 3 Results

### 3.1 Observation of Scaffold Materials

**FIGURE 1:**
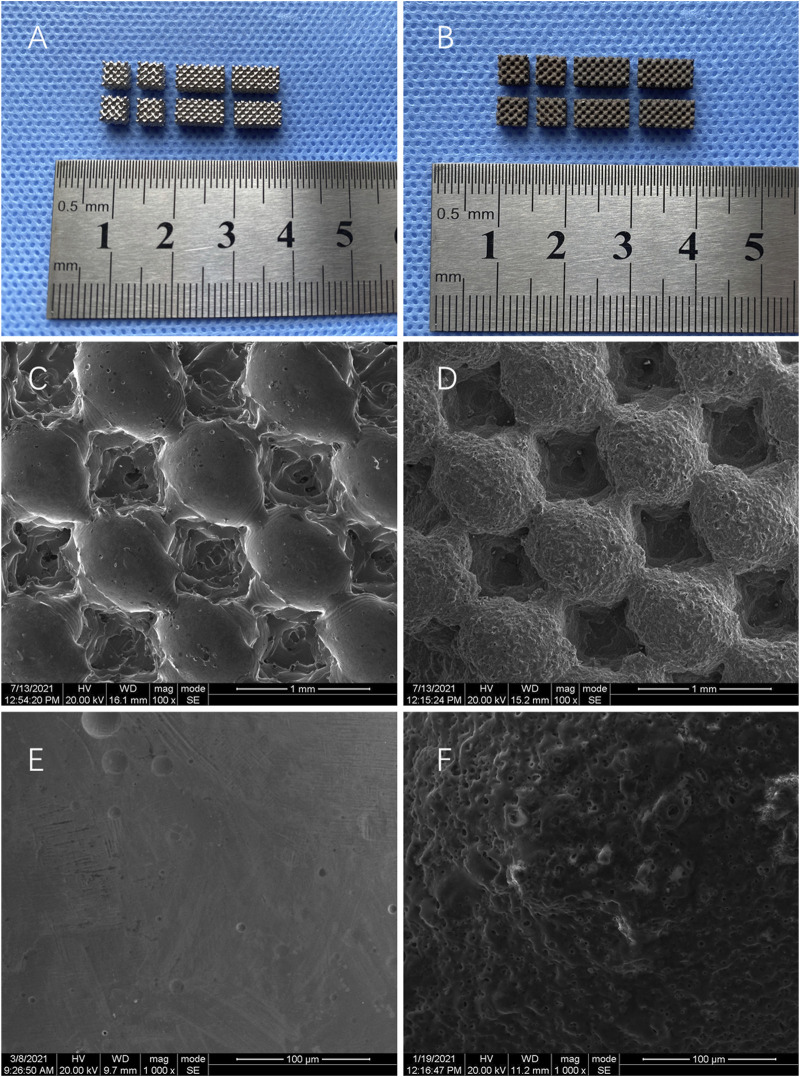
General observation: **(A)** Ti; **(B)** Gr-Ti; SEM observation: **(C,E)** Ti; **(D,F)** Gr-Ti.

The results showed that the surface of Ti is smooth and metallic (Figure 1A). In contrast, Gr-Ti has a gray-black surface, no metallic luster, rough surface, and tightly bonded coating without shedding (Figure 1B). SEM observation showed that the surface of the Ti scaffold was smooth, and the shape rules were consistent with the design (Figures 1C,E). The surface of Gr-Ti is rough, with good phase continuity and no cracks, and there are certain pores on the coating surface (Figures 1D,F).

Furthermore, the porosity was 69 ± 3% and the pore size was 546 ± 21 μm. The results of surface composition analysis show that the peak value of the carbon element can be seen in the energy spectrum analysis of the Gr-Ti composite coating material, indicating that the surface elemental composition of the scaffold material primarily comprised of the carbon contained in the graphene coating, which can prove the reliability of the existence of coating. These results were described in more detail in our previous study.

### 3.2 Morphological Observation and Identification of Adipose Stem Cells-Exos

**FIGURE 2:**
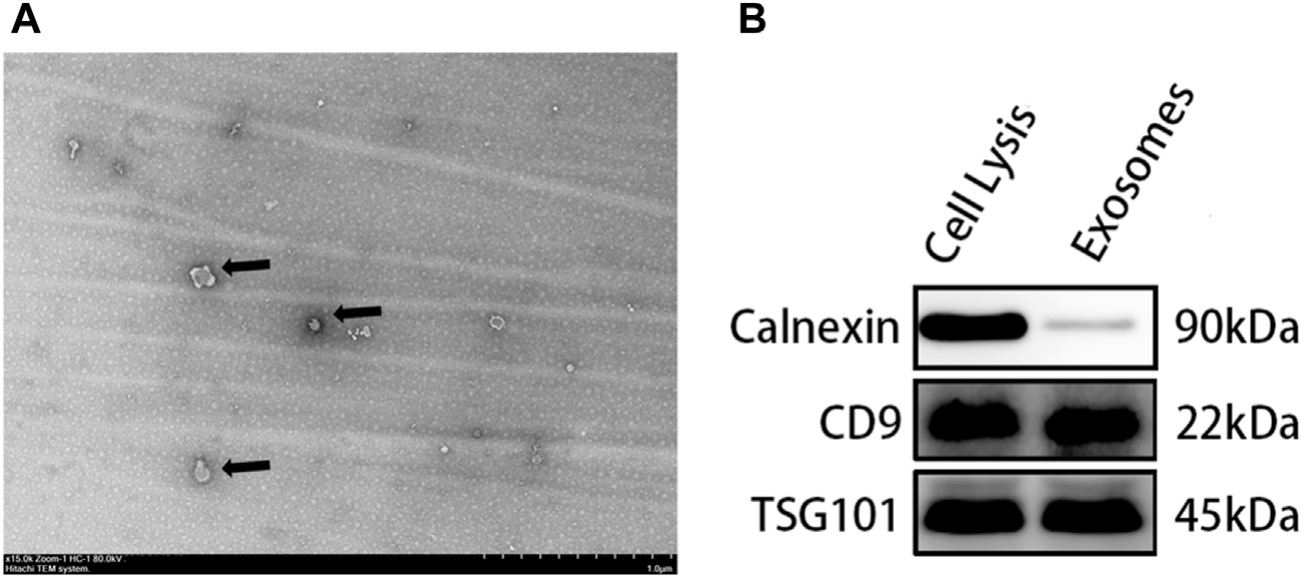
Identification of Exos: **(A)** The size and morphology of exosomes observed using TEM; **(B)** Specific markers of exosomes detected by Western blot.

The TEM results showed that the corresponding ADSC-Exos were nearly round in general. Furthermore, the edge was clear, the content density was low, and the diameter was between 30 and 150 nm. Western blot showed positive expression of proteins CD9 and TSG101, but almost no expression of the endoplasmic reticulum protein calnexin Figure 2.

### 3.3 Adhesion of Adipose Stem Cells to Gr-Ti Scaffold

**FIGURE 3:**
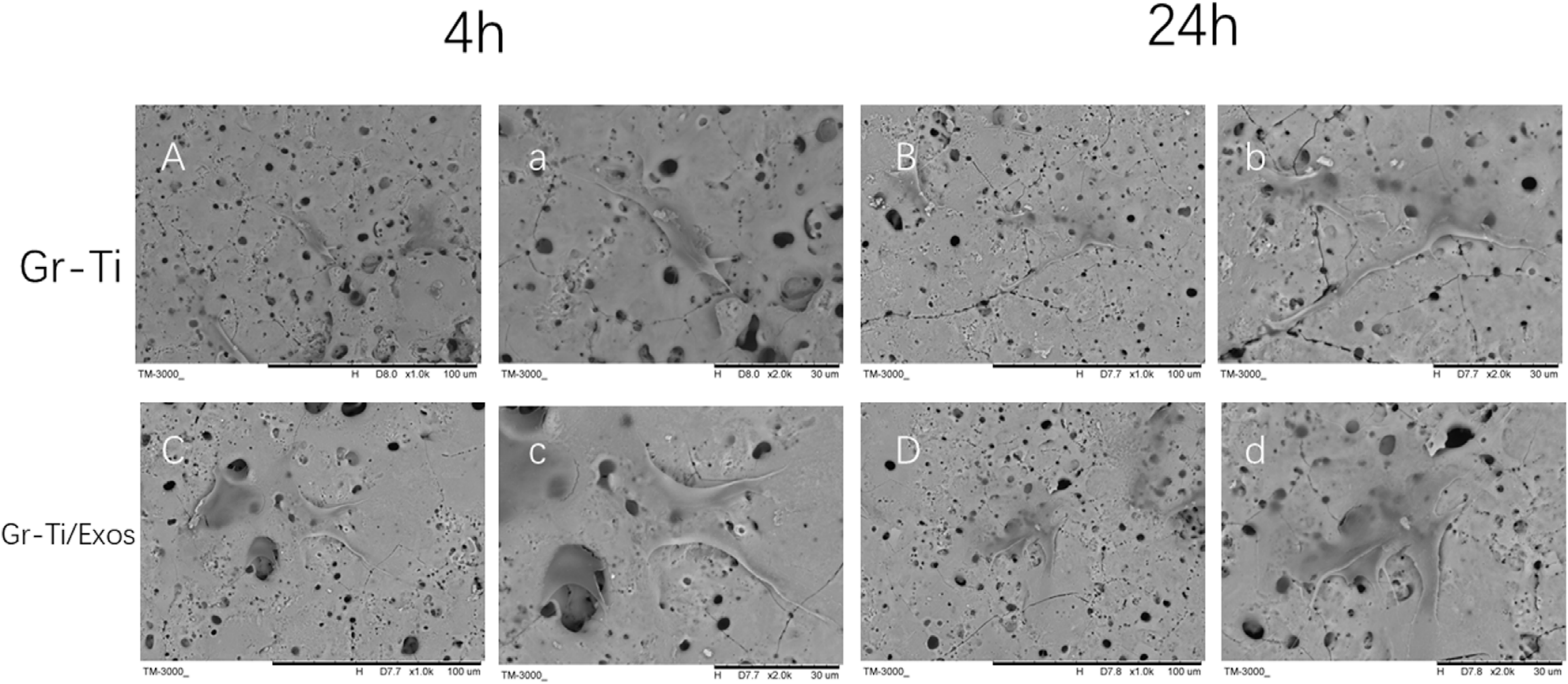
Adhesion of ADSCs to scaffolds (SEM): The morphology of ADSCs in Gr-Ti/Exos group were better than that in Gr-Ti group. [**(A–D)** × 1,000; **(a–d)** × 2,000].

**FIGURE 4:**
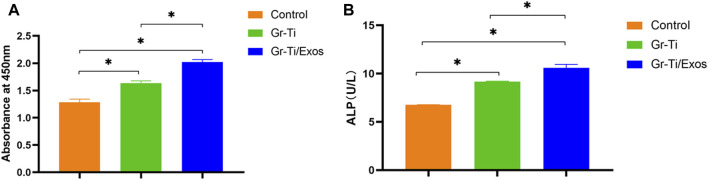
**(A)** CCK-8 detection results: the number of ADSCs in the Gr-Ti/Exos group was significantly higher than that in the Gr-Ti group; **(B)** ALP activity test results: ALP activity in the Gr-Ti/Exos group was higher than that of the Gr-Ti group (*n* = 5, ^∗^ *p* < 0.05).

As seen in Figure 4, during the early stage of culture at ∼4 h, the morphology of the ADSCs in the Gr-Ti scaffold group was similar without significant difference regardless of the influence of Exos. Scattered round or oval ADSCs adhesion could be seen on the scaffold surface, and granular bulges were visible on the surface of ADSCs. Moreover, a small amount of granular extracellular matrix can be seen around the cells (Figures 3Aa,Cc). After 24 h of culture, in the Gr-Ti/Exos group, the peripherally extended pseudopodia of the cells on the scaffold significantly extended and grew across the pores of the scaffold, forming an anchor structure that firmly bonded with the scaffold and connected with the pseudopodia of other cells, while the cells joined into slices (Figures 3Dd). This characteristic was not obvious in the Gr-Ti group, with atypical morphology (Figures 3Bb).

### 3.4 Detection of Cell Proliferation and Osteogenic Activity

As shown in Figure 4A, ADSCs were incubated in medium with 50 μl Exos (Gr-Ti/Exos group) or without Exos (Gr-Ti group) for 24 h, respectively. The results showed that the number of ADSCs in the Gr-Ti/Exos group was significantly higher than that in the Gr-Ti group (*p* < 0.05), indicating that Exos can promote the proliferation of ADSCs in the Gr-Ti scaffold.

ADSCs were co-cultured with the Gr-Ti/Exos group and Gr-Ti group for 24 h, respectively. The results shown in Figure 4B, indicated higher ALP activity in the Gr-Ti/Exos group than that of the Gr-Ti group (*p* < 0.05), suggesting that Exos could promote the osteogenic differentiation of ADSCs on Gr-Ti scaffolds.

### 3.5 Expression of Osteogenic Genes Detected by Quantitative Real-Time PCR

**FIGURE 5:**
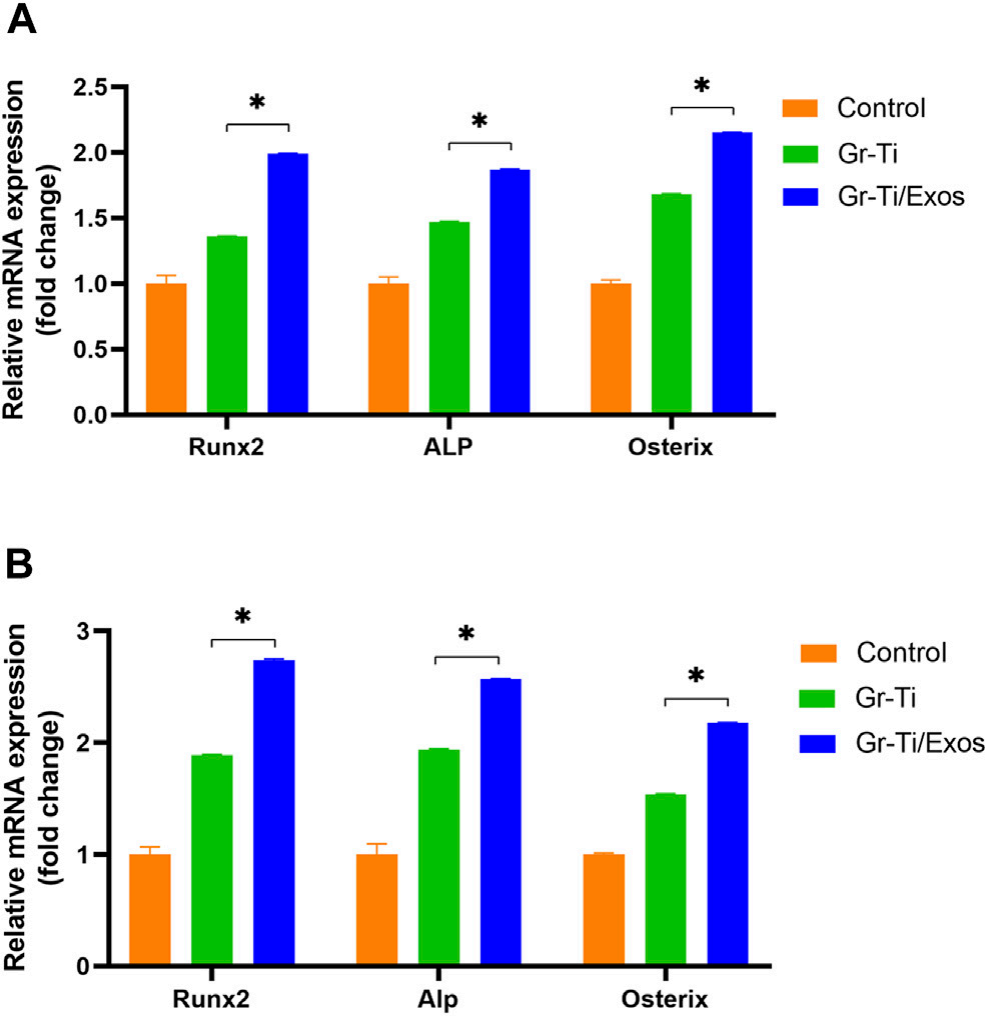
Expression of osteogenic genes: **(A)** Osteogenic induction for 7 days; **(B)** Osteogenic induction for 14 days: the mRNA levels of RUNX2, ALP, and Osterix in the Gr-Ti/Exos group were significantly higher than those in the Gr-Ti group at both time points (^∗^ *p* < 0.05).

To detect the expression of *RUNX2*, *ALP*, and *Osterix* genes related to Exos promoting the osteogenic differentiation of ADSCs, qPCR was used to detect the mRNA levels after 7 and 14 days of incubation, respectively. As shown in Figure 5, the mRNA levels of *RUNX2*, *ALP*, and *Osterix* in the Gr-Ti/Exos group were significantly higher than those in the Gr-Ti group at both time points (*p* < 0.05), suggesting that exosomal Gr-Ti scaffold can promote the osteogenic differentiation of ADSCs.

### 3.6 Levels of Osteogenic-Related Proteins Detected by Western Blot

**FIGURE 6:**
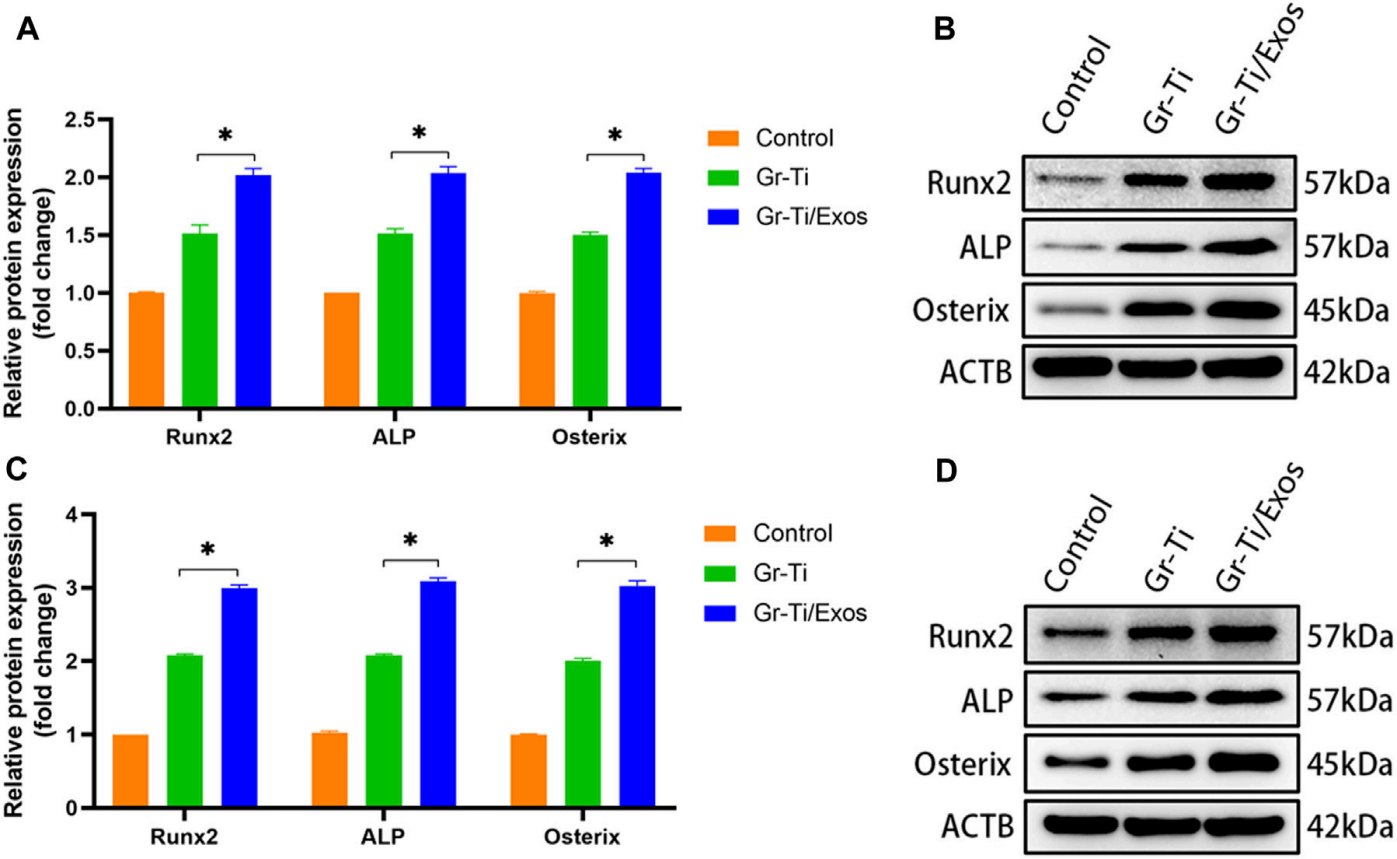
Expression of osteogenic-related protein: **(A,B)** after osteogenic induction for 7 days; **(C,D)** after osteogenic induction for 14 days: the levels of RUNX2, ALP, and Osterix in the Gr-Ti/Exos group were higher than those in the Gr-Ti group at 7 and 14 days (^∗^ *p* < 0.05).

To further verify that the Gr-Ti scaffold combined with Exos promoted the osteogenic differentiation of ADSCs, the protein levels of ADSC osteogenic differentiation-related RUNX2, ALP, and Osterix were detected. After 7 and 14 days of incubation (Figure 6), the levels of RUNX2, ALP, and Osterix in the Gr-Ti/Exos group were higher than those in the Gr-Ti group at 7 and 14 days (*p* < 0.05), respectively, indicating that Exos can promote the osteogenic differentiation of ADSCs on the Gr-Ti scaffold.

### 3.7 Gr-Ti/Exos Composite Scaffolds Promote Osteogenic Differentiation of Adipose Stem Cells Through Wnt Signaling Pathway

**FIGURE 7:**
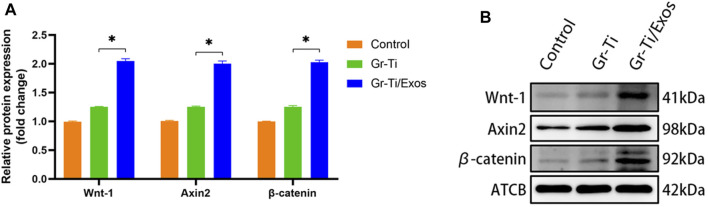
Wnt pathway-related protein levels: **(A)** Relative intensity analyses of Western blot results; **(B)** The expression of protein analysed by Western blot. The level of Wnt-1, Axin2, and β-catenin in the Wnt signaling pathway in the Gr-Ti/Exos group were higher than those in the Gr-Ti group (^∗^ *p* < 0.05).

Western blot was used to detect the levels of key proteins Wnt-1, Axin2, and β-catenin in the Wnt signaling pathway. It was found that after 7 days of osteogenic induction (Figure 7), the level of each protein in the Gr-Ti/Exos group was higher than that in the Gr-Ti group (*p* < 0.05).

**FIGURE 8:**
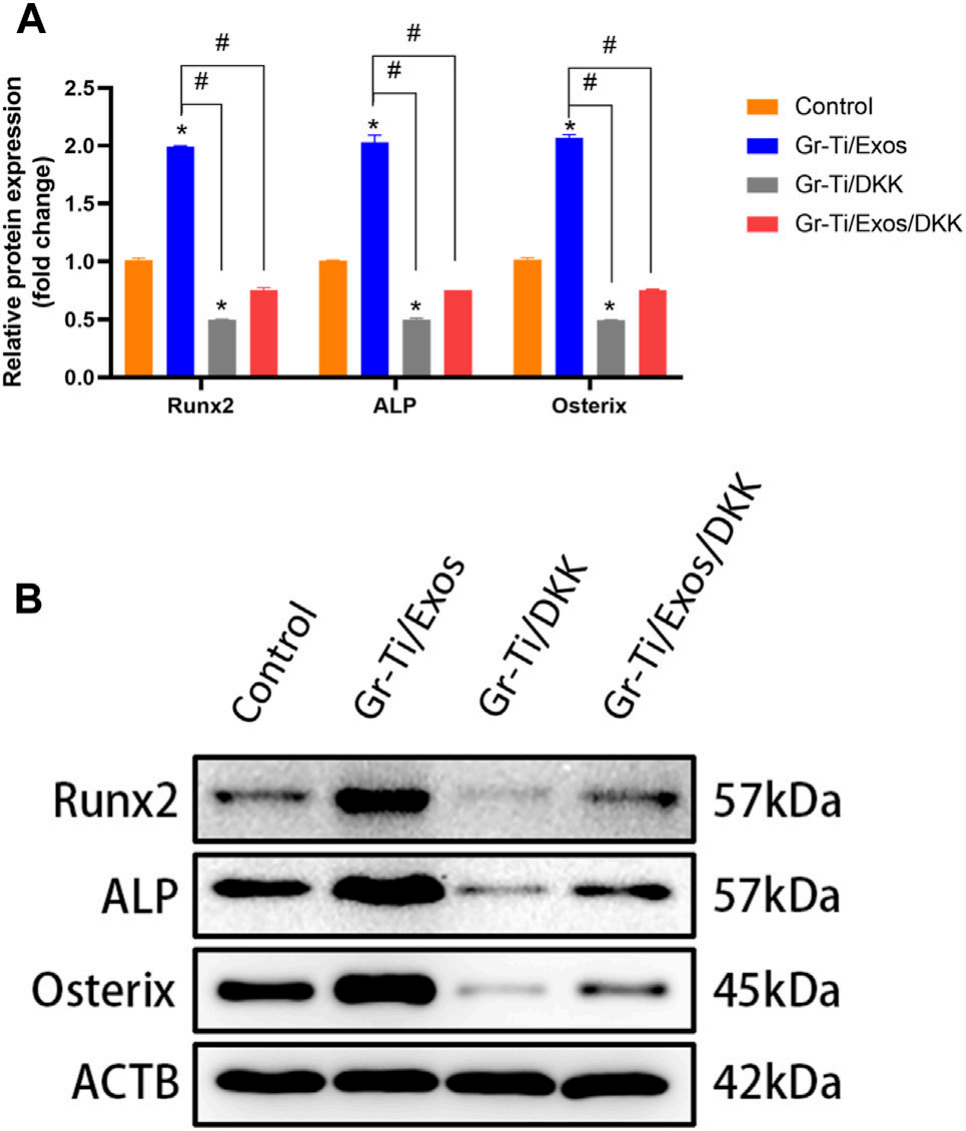
Levels of osteogenic proteins after addition of Wnt pathway inhibitor DKK. **(A)** Relative intensity analyses of WB results; **(B)** The expression of protein analysed by WB. (^∗^represents statistical difference between control and other groups; ^#^indicates statistical difference between Gr-Ti/Exos, DKK, and Gr-Ti/Exo/DKK groups; ^∗^ *p* < 0.05; ^#^ *p* < 0.05).

**FIGURE 9:**
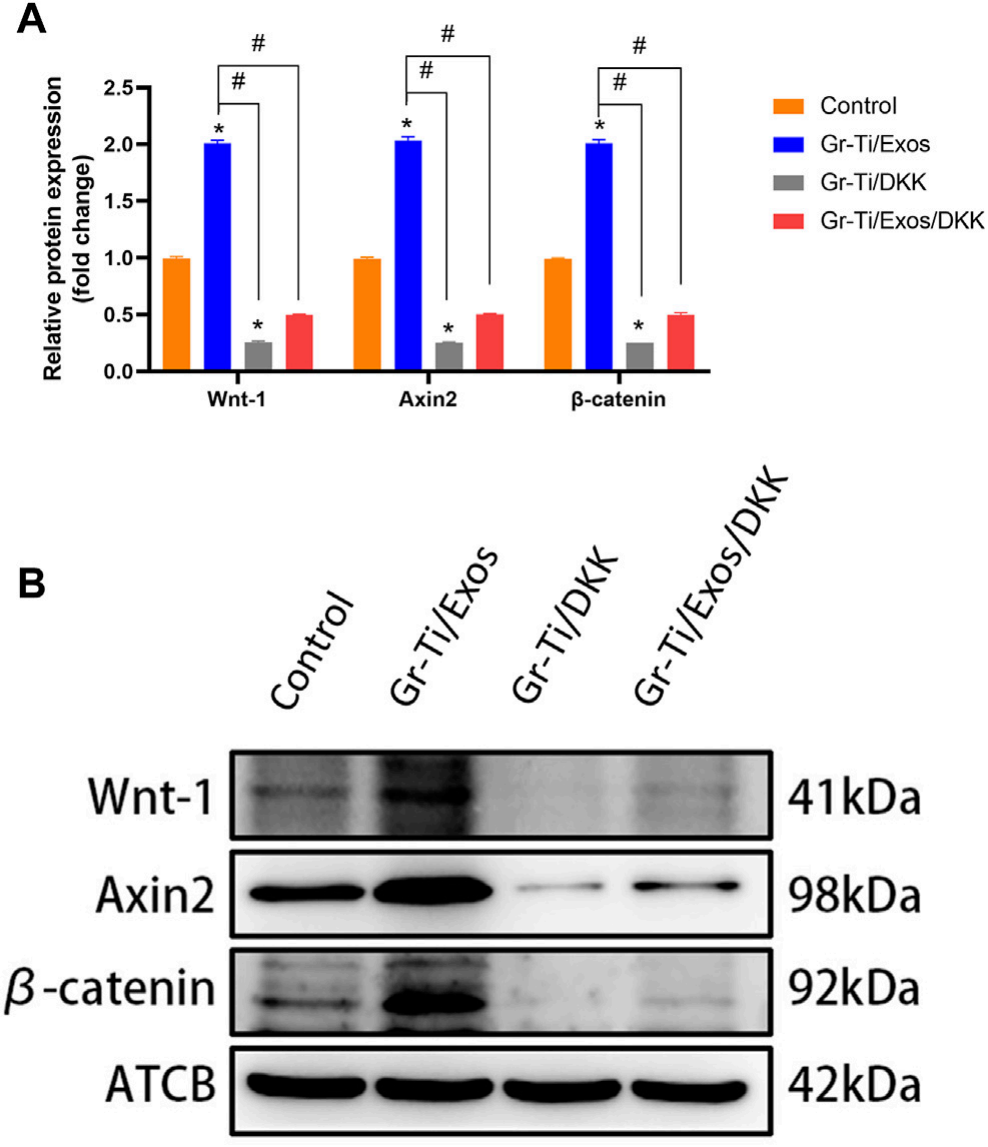
Key proteins of the Wnt pathway after addition of Wnt pathway inhibitor DKK. **(A)** Relative intensity analyses of WB results; **(B)** The expression of protein analysed by WB. (^∗^represents statistical difference between control and other groups; ^#^indicates statistical difference between Gr-Ti/Exos, Gr-Ti/DKK, and Gr-Ti/Exos/DKK groups; ^∗^ *p* < 0.05; ^#^ *p* < 0.05).

To further verify the specific molecular mechanism of Exos-regulation of osteogenic differentiation in ADSCs, the Wnt signaling pathway inhibitor DKK group was added. The levels of osteogenic-related proteins RUNX2, ALP, and Osterix as well as the key proteins of the Wnt signaling pathway (Wnt-1, Axin2, and β-catenin) were detected again. The results showed (Figures 8, 9) that there was no significant difference in protein levels between the Gr-Ti/Exos/DKK and control groups (*p* > 0.05), and that of the Gr-Ti/Exos and Gr-Ti/DKK groups were significantly different from the control group (*p* < 0.05), while the levels of the Gr-Ti/Exos group was significantly higher than that of the control group. Furthermore, the levels of the related proteins were significantly decreased after adding inhibitor DKK (*p* < 0.05).

### 3.8 Mandible Defect Repair in Rabbits

#### 3.8.1 General Observation

**FIGURE 10:**
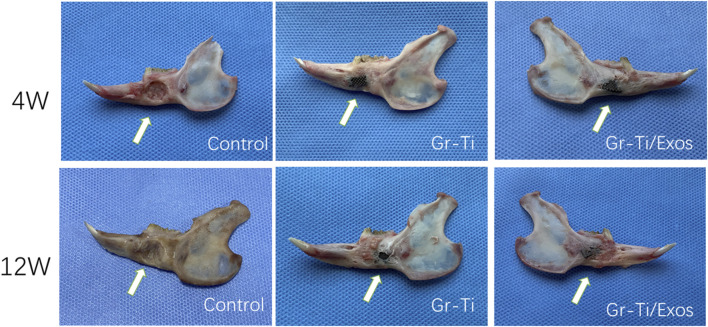
General observation of scaffold materials.

At 4 weeks post-surgery, there was no shift in the position of implanted materials of the Gr-Ti and Gr-Ti/Exos groups, the surface of the material was covered by fibrous connective tissue, and the implants were loosely bound to the edge of the defect area, with no significant difference between the two groups. In the blank control group, the bone defect cavity began to grow granulation tissue. At 12 weeks post-surgery, the implants in the Gr-Ti and Gr-Ti/Exos groups were firmly connected to the edge of the defect, while the implants in the Gr-Ti group were covered by new bone, which was soft and became transparent when pressed. In the Gr-Ti/Exos group, the defect boundary disappeared, and the surface was almost completely covered by new bone. The new bone was hard and became imperceptible when pressed. In the blank control group, the bone defect cavity was filled with granulation tissue, and the defect area was not significantly reduced (Figure 10).

#### 3.8.2 Micro-CT Detection

**FIGURE 11:**
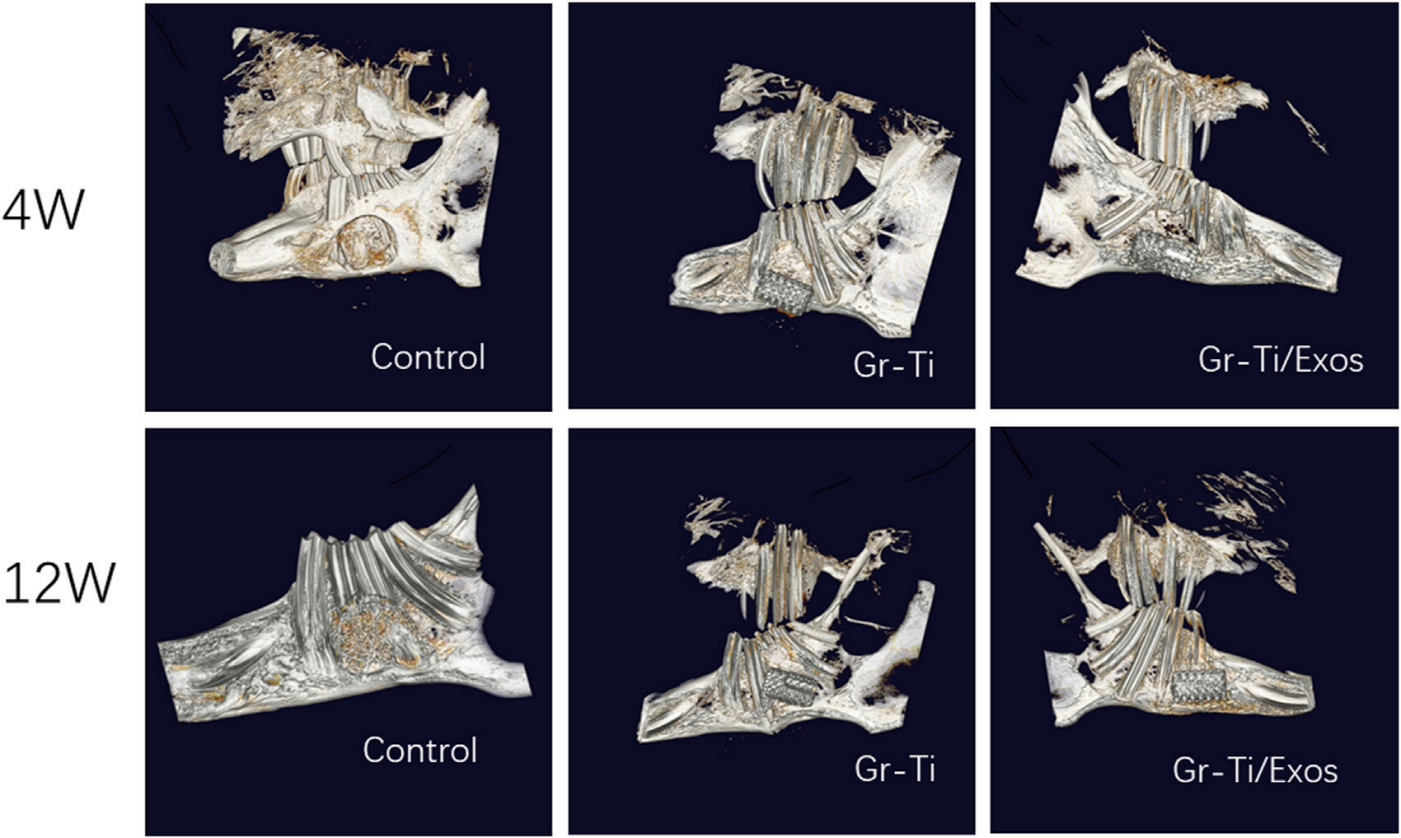
Computerized tomography (CT) images after 4 and 12 weeks.

CT images (Figure 11) showed that although a vast amount of new bone was generated in the Gr-Ti group at 12 weeks after surgery, most of the new bone existed at the edge of the defect area. Although the defect area was reduced, it was not enough to cover the whole defect area. Metal artifacts were seen locally, indicating that some metal materials were still exposed. In the Gr-Ti/Exos group, the bone defect area was significantly reduced 12 weeks after surgery, and there was no obvious metal artifact around the bone tissue. The bone tissue density was similar to that of the original bone tissue, and the new bone was closely bound to the surrounding, indicating that the bone formation ability was better than that of the Gr-Ti group. In the blank control group, there was still no obvious new bone formation 12 weeks after surgery, and all the bone callus in the defect area were loose structure, which failed to heal by itself.

#### 3.8.3 Bone Mineral Density and Biomechanical Determination

**FIGURE 12:**
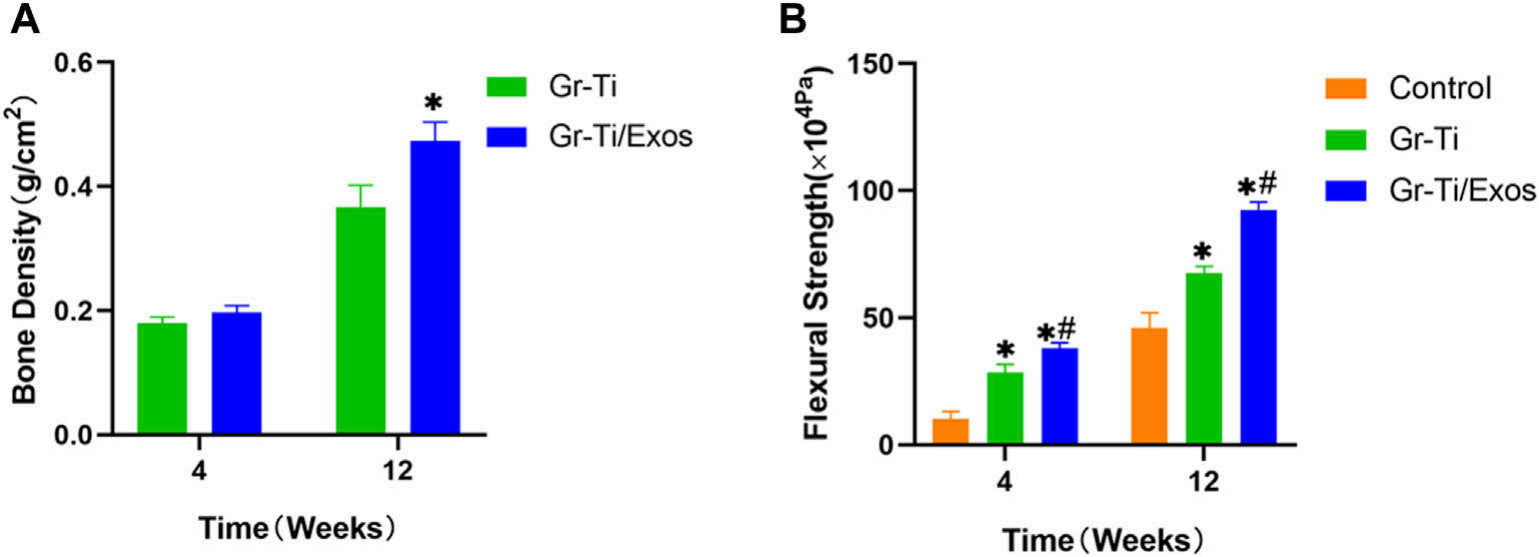
**(A)** Bone density measurement; **(B)** Biomechanical measurement (^∗^represents the comparison between the stent and blank control groups, *p* < 0.05; ^#^represents the comparison between the Gr-Ti/Exos and Gr-Ti groups, *p* < 0.05).

The local bone mineral density of the mandibular defect was measured by a dual-energy X-ray bone density analyzer at 4 and 12 weeks after implantation of the scaffold material to predict the calcification of the mandible (Figure 12A). The blank control group had no obvious new bone formation, and no clear bone density could be detected. After 4 weeks, bone mineral density increased in both the Gr-Ti and Gr-Ti/Exos groups, but there was no significant difference between the two groups (*p* > 0.05). The bone mineral density of the Gr-Ti/Exos group (0.47 ± 0.03 g/cm^2^) was significantly higher than that of the Gr-Ti group (0.36 ± 0.04 g/cm^2^) (*p* < 0.05).

As shown in Figure 12B, we measured the bending strength of each material at weeks 4 and 12. Over time, the bending strength of each group increased gradually. At 4 weeks after implantation, the bending strength of the Gr-Ti stent and Gr-Ti/Exos groups (28.60 ± 3.09 × 10^4^ Pa and 38.05 ± 2.12 × 10^4^ Pa) was significantly higher than that of the control group (10.20 ± 3.04 × 10^4^ Pa) (*p* < 0.05). At 12 weeks after implantation, the bending strength of the Gr-Ti/Exos group was 92.43 ± 3.12 × 10^4^ Pa, which was significantly higher than the 67.70 ± 2.43 × 10^4^ Pa of the Gr-Ti group and the 46.04 ± 5.89 × 10^4^ Pa of the control group (*p* < 0.05).

#### 3.8.4 Histological Observation

At 4 weeks after surgery, a small amount of new bone tissue was formed at the edge of the scaffold material in both the Gr-Ti and Gr-Ti/Exos groups. In the Gr-Ti group, the boundary between the scaffold and bone defect area was still clear, and no obvious bone trabecula was formed. In the Gr-Ti/Exos group, new bone grew into the edge of the bone defect area, parts of the bone trabeculae were interconnected, and the distribution of red blood cell fiber tissue was found in the pores. In the blank control group, the edge of the bone defect area was still clear, no obvious new bone tissue was formed, and was filled with only fibrous connective tissue.

**FIGURE 13:**
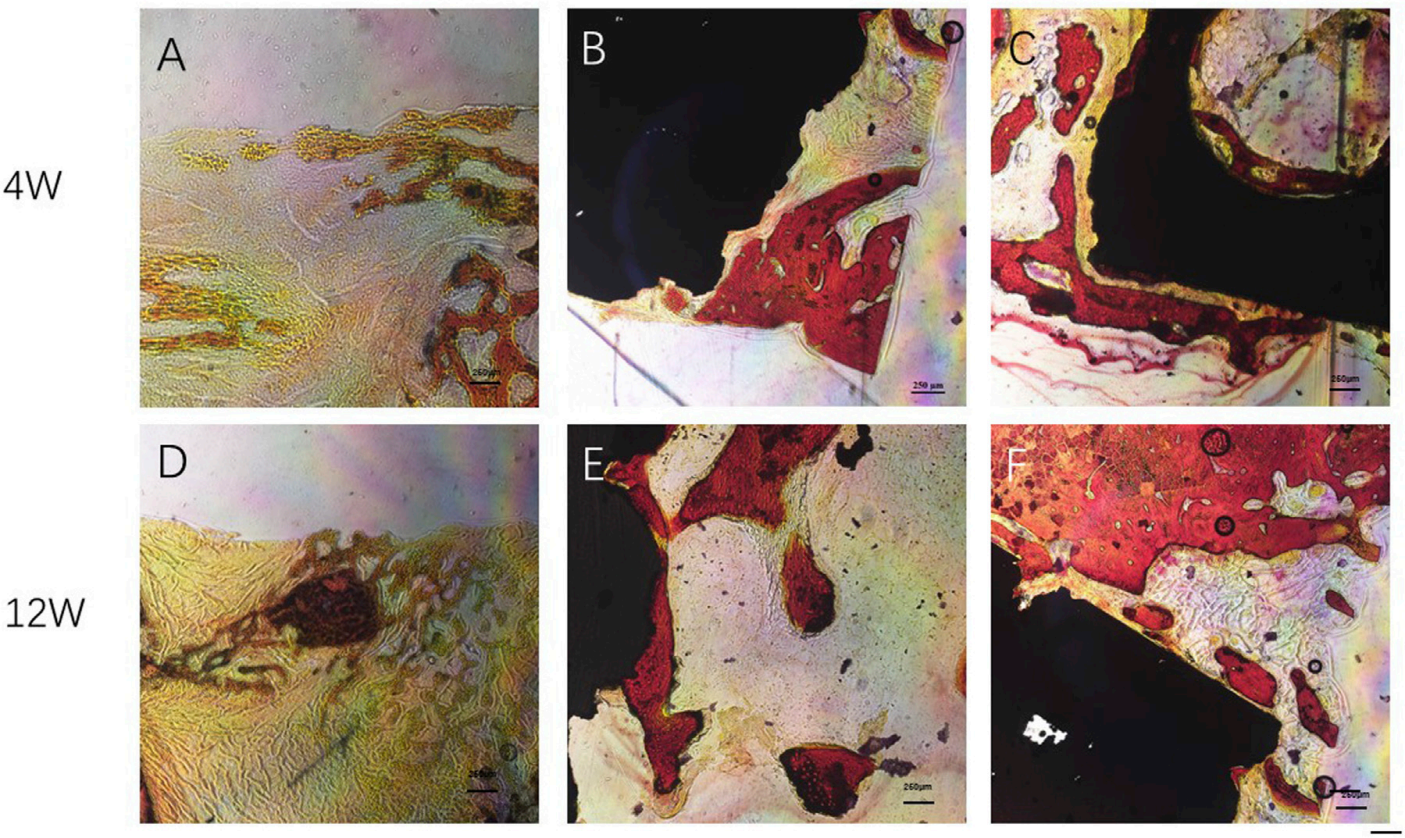
Van Gieson staining: Newborn bone tissue is stained red. **(A,D)** Control group; **(B,E)** Gr-Ti group; **(C,F)** Gr-Ti/Exos group: a significant amount of new bone tissue grew into the bone defect area and material pores of the Gr-Ti/Exos group.

At 12 weeks after surgery, a significant amount of new bone tissue grew into the bone defect area and material pores of the Gr-Ti/Exos group. This new bone tissue was dense, and the boundary of the defect area became unclear. Compared with the Gr-Ti/Exos group, the margin of bone defect was significantly higher in the Gr-Ti/Exos group, and the new bone was less than that in the Gr-Ti/Exos group, although the new bone formed was greater than that formed by the fourth week. No obvious new bone tissue was found in the blank control group, and the bone defect was filled with a lot of connective tissue Figure 13.

## 4 Discussion

At present, the development and improvement of traditional titanium alloy are undergoing a revolution. Since the performance of most implants is dependent on their surface properties, using the principles of engineering to improve surface characteristics is one of the most appropriate strategies for developing next-generation titanium implants (Yi et al., 2022).

In this study, the results showed that porous titanium alloy material with a three-dimensional pore structure could effectively reduce the elastic modulus of implants to match normal bone tissue, and provided support for the interlocking between bone tissue and implants to improve the stability of the implant (Fousová et al., 2017). Some studies have found that at a porosity of 70%, the elastic modulus of titanium alloy can be reduced to 0.7 GPa which almost eliminated the stress shielding (Jing et al., 2020). In addition, some researchers have manufactured titanium alloy materials with an elastic modulus of 1 GPa and porosity of 85% by the SLM method, which achieved relatively high mechanical properties to meet the application requirements of bone repair (Zheng et al., 2020). Here, the optimization of the porous titanium alloy implant rod and the results show that the implant had a large pore size (550 μm) and high porosity. According to micro-CT detection, the porous titanium alloy formed by SLM processing had good properties, with full through-through pores and corresponding porosity of 69% ± 3%, which meets the design standards of pore structure and pore parameters.

Since its discovery, graphene has been widely used in biomedical surface engineering due to its unique physical and chemical properties as well as biocompatibility (Xie et al., 2019; Ma et al., 2020). Graphene can improve the surface activity of biomaterials without changing the properties of the biomaterial matrix (Zhu et al., 2020). Graphene is biologically more stable than other two-dimensional nanomaterials, such as Phosphorene or the Borophene (Tatullo et al., 2019a; Tatullo et al., 2019b). Therefore, in our previous study, we designed and manufactured a porous titanium alloy scaffold with a composite graphene coating (Sun et al., 2021). Concomitantly, the role of stem cells in promoting tissue repair and regeneration through the paracrine pathway has received increasing attention. Therefore, in this study, we focused on the safety and possible mechanism of ADSC-Exos and composite graphene-coated porous titanium alloy scaffolds in bone tissue regeneration.

Exos are types of EVs derived from endosomes secreted by MSCs, lymphocytes, and epithelial cells. All EVs are cup-shaped, and the respective identification of Exos, microvesicles, or apoptotic bodies depends mainly on the mode of generation, diameter, and surface markers (Popa and Stewart, 2021). At a diameter of 30–150 nm, the gold standard for Exo identification is transmission electron microscopy (Andreu and Yáñez-Mó, 2014). The exosome lipid-bilayer membrane has characteristic surface markers, such as the membrane-binding proteins CD9, CD63, and TSG101, which can be readily identified by western blot. In this study, after deriving Exos from ADSCs by an ultra-high-speed differential centrifuge method, their characteristic cup-shaped appearance and diameter were observed *via* SEM. Western blot results showed that the protein levels of CD9 and TSG101 on Exos were positive, while the corresponding calnexin was negative, which could be used to distinguish the Exos from the ADSCs.

Good cell adhesion is characterized by fusiform or fusiform appearance and extension of pseudopodia. It could be seen from early cell adhesion experiments that, after the addition of Exos, the growth morphology and number of ADSCs in the Gr-Ti scaffold were better than those on the scaffold without Exos, indicating that the Gr-Ti loaded with Exos could better promote cell adhesion. This may be a result of constructing a three-dimensional structure similar to the microenvironment *in vivo*—i.e., it can accommodate a greater number of cells than traditional two-dimensional cultures, while retaining a large amount of the outer matrix around the cells.

Whether the seed cells can maintain and promote their viability after being inoculated into the scaffold material is an important criterion to qualify the scaffold material. By comparing the CCK-8 and ALP detection results of the Gr-Ti and Gr-Ti/Exos groups, we found that the number and osteogenic index of the scaffolds with Exos were higher than those without Exos, proving that Exos significantly promoted the growth of ADSCs in the Gr-Ti scaffolds.

In bone tissue engineering, good adhesion of seed cells to scaffold materials is a prerequisite for cell proliferation and osteogenic differentiation, while the influence of scaffold materials on the osteogenic differentiation of stem cells requires further evaluation at the molecular level. ALP, osteocalcin, and type I collagen can be used to determine the conversion of stem cells into osteoblasts. RUNX2 is also closely associated with osteogenic differentiation (Maqsood et al., 2020). Studies have shown that rat ADSC-Exos can promote the adhesion and proliferation of BMSCs, enhance the activity of ALP, upregulate the expressions of *RUNX2* and *ALP* genes, and promote the osteogenic differentiation of stem cells (Nikfarjam et al., 2020). Martins et al. (2016) found that mediums that activated transcription and *Runx2* could induce osteogenesis of MSCs, and their secreted Exos could better upregulate the expression of osteogenic marker genes such as *ALP*, *BMP2*, and *SP7*, effectively promoting osteogenic differentiation of MSCs. In addition, it was found during osteoblast differentiation, the MSC-Exos can promote the expression of *TGF-β1*, *RUNX2*, and *OSX*. Here, our PCR and western blot analysis showed that the expression of *RUNX2*, *ALP*, and *OSX* osteogenic genes and proteins in the Gr-Ti/Exos group was significantly increased after the addition of Exos into the scaffold material, which was significantly different from that in the Gr-Ti and control groups without Exos. These results suggest that Gr-Ti loaded with Exos can promote osteogenic differentiation in ADSCs.

Wnt signaling is closely related to biological development and plays a regulatory role in biological processes such as embryo development and tissue regeneration (Haffner-Luntzer, 2021). Wnt signaling pathway mainly consists of the Wnt family, such as CK1, GSK-3β, APC, and Axins (Koziński and Dobrzyń, 2013). Studies have shown that graphene has good osteogenic induction and can accelerate osteogenic differentiation by activating the Wnt/β-catenin-related signaling pathways (Wang et al., 2020). β-catenin is an important node factor in this signaling pathway, which can regulate the *RUNX2* gene and promote osteogenic differentiation of ADSCs when activated by Wnt signaling (Zhang et al., 2015; Lu et al., 2017). Researchers also found that Exos can increase the expression of Wnt signaling pathways *Wnt-1*, *Axin2*, and *β-catenin* (Gu et al., 2019; Yang et al., 2020). Wnt/β-catenin signaling pathway inhibitor DKK can bind to the LRP5 receptor, competitively inhibit the Wnt ligand-protein, reduce β-catenin level, and then inhibit osteogenic differentiation. In this study, when we added the Wnt pathway inhibitor DKK, the expressions of *Wnt1*, *Axin2*, and *β-catenin* were significantly decreased, and the levels of osteogenic-related proteins RUNX2, ALP, and Osterix were also decreased, suggesting that the role of Exos in promoting ADSCs osteogenesis could be inhibited by DKK. Therefore, it was indirectly shown that Exos can mediate the osteogenic differentiation of ADSCs through the Wnt/β-catenin signaling pathway.

At present, there are few *in vivo* experimental studies on porous titanium alloy scaffolds, especially studies on the combination of graphene surface modification and Exos. The final application of any kind of material needs *in vivo* verification to confirm its safety, reliability, and effectiveness. Furthermore, the establishment of an animal bone defect model must meet the requirement of a critical bone defect (Machavariani et al., 2019). In this study, a rabbit model of full-layer bone defect of mandible was characterized by clear anatomical location, superficial location, simple approach, easy operation, and high success rate. For the experiment, a 0.8 cm full-thickness defect of the mandible was inflicted in the blank control group, which was filled with a large amount of fibrous tissue and granulation tissue 12 days after the operation, showed no obvious new callus formation, and did not meet the clinical bone healing standard but did meet the standard definition of a critical bone defect. This model preparation method was safe, reliable, and reproducible, providing an effective way to evaluate the bone-binding ability of scaffold materials.

By general observation and micro-CT results, it can be seen that there were large low-density shadows around the materials in both the Gr-Ti/Exos and Gr-Ti groups 4 weeks after surgery, and these low-density shadows decreased in both groups 12 weeks after surgery, indicating bone defects near implants were repaired as time extended. Concurrently, the bone tissue of the Gr-Ti/Exos group encompassed the scaffold material and bone defect area, and there was no obvious boundary between the scaffold material and the surrounding area, indicating that the bone composition effect of the Gr-Ti/Exos group was better than that of the Gr-Ti group.

Through bone growth volume fraction and bone mineral density measurement, it was found that the bone volume fraction of the Gr-Ti/Exos group was significantly higher than that of the Gr-Ti group at 12 weeks after surgery, indicating a higher number of new bone tissue in the material, which was also consistent with the bone mineral density measurement results. Comparison of these two groups suggests that Exos have a definite promoting effect on the osteogenic differentiation of ADSCs on composite graphene-coated porous titanium alloy scaffolds.

The mechanical test results showed that the flexural strength of the scaffold group was higher than that of the blank control group at the 4th and 12th week after surgery, indicating that the scaffold material can significantly reduce the stress shielding of the material itself and its mechanical properties can adapt to normal bone tissue. Meanwhile, the flexural strength of the Gr-Ti/Exos group was superior to that of the Gr-Ti group, indicating that the Exos play a key role in the bone tissue healing process and can better promote the osteogenic differentiation of ADSCs. These results provide mechanical experimental support for the application of Exos in the field of bone tissue engineering.

Histological staining showed that in the scaffold group, the new bone tissue was found around the defect area at 4 weeks after surgery; however, the gaps between the scaffold and bone tissue were not yet closed. At the 12th week after surgery, the amount of new bone that covered the defect area in the Gr-Ti/Exos group was significantly better than that in the Gr-Ti group, and there was no obvious gap between the new bone and scaffold, showing a good bone integration effect. In contrast, in the blank control group, there was only scattered fibrous tissue filling in the defect area, and no obvious new bone tissue was formed.

In summary, we combined ADSCs as “seed cells” and Exos as “inducers” on a Gr-Ti scaffold for bone tissue engineering, to show its prospective application in repairing maxillofacial bone defects. *In vitro* experiments have shown that the Gr-Ti scaffolds have good safety and biocompatibility, and can promote the adhesion and osteogenic differentiation of ADSCs. *In vivo* and *in vitro* experiments showed that ADSC-derived Exos can promote the growth of ADSCs in the Gr-Ti scaffold, and upregulate the expression of osteogenic genes and proteins, which may be related to the Wnt signaling pathway. However, the mechanism of Exos promoting the osteogenic differentiation of ADSCs remains to be further studied. Our ultimate goal is to achieve the clinical application of this method, so further research is needed in the immunogenicity of stem cells, biocompatibility of scaffold materials, standardization of extraction and preservation of Exos and other aspects.

## 5 Conclusion

In this study, we first showed that Gr-Ti scaffolds have good biocompatibility, which can promote the adhesion and osteogenic differentiation of ADSCs. Second, Exos derived from ADSCs further promoted the osteogenic differentiation of ADSCs in Gr-Ti scaffolds. Third, the mechanism of Exos in promoting osteogenic differentiation of ADSCs is related to the Wnt signaling pathway. Lastly, Gr-Ti scaffolds with ADSCs and ADSC-derived Exos successfully repaired rabbit mandibular defects, laying a foundation for the clinical application of bone tissue engineering in repairing irregular bone defects in the maxillofacial region. However, this paper only observed some phenomena of the joint action of Gr-Ti scaffold and Exos to promote bone tissue regeneration, and its deep mechanism needs to be further studied and explored, which is also the direction that the authors need to study in the future.

## Data Availability

The original contributions presented in the study are included in the article/Supplementary Material, further inquiries can be directed to the corresponding authors.

## Abbreviations

ADSCs, adipose stem cells; ANOVA, analysis of variance; CT, computerized tomography; DKK, dickkopf; EVs, extracellular vesicles; Exos, Exosomes; Gr-Ti, graphene-composite titanium alloy scaffold; MAO, micro-arc oxidation; MSCs, mesenchymal stem cells; SEM, scanning electron microscopy; SLM, selective laser melting.
